# P-1326. Prevalence of Rectal Carbapenem Resistant Enterobacterales (CRE) Carriage Among Patients Attending Primary, Secondary and Tertiary Healthcare Facilities in West Africa

**DOI:** 10.1093/ofid/ofaf695.1514

**Published:** 2026-01-11

**Authors:** Olukemi A Adekanmbi, Oluwafemi O Popoola, Sulaiman Lakoh, Adeola Fowotade, Ini Adebiyi, Umu Barrie

**Affiliations:** University College Hospital, Ibadan, Ibadan, Oyo, Nigeria; College of Medicine, University of Ibadan, Ibadan, Oyo, Nigeria; College of Medicine and Allied Health Sciences, University of Sierra Leone, Freetown, Western Area, Sierra Leone; College of Medicine, University of Ibadan, Ibadan, Oyo, Nigeria; University College Hospital, Ibadan, Ibadan, Oyo, Nigeria; Infectious Disease Research Network, Freetown, Western Area, Sierra Leone

## Abstract

**Background:**

Carbapenem Resistant *Enterobacterales* (CRE) are responsible for life threatening antimicrobial resistant (AMR) infections in developing countries. They colonize the gastrointestinal tract and spread due to poor infection prevention and control (IPC) practices. CRE infections are difficult to treat in these settings because of limited access to effective antibiotics. The West Africa Region is reported to have the highest burden of AMR infections in sub-Saharan Africa and our study aims to describe the rectal carriage of CRE amongst patients attending healthcare facilities in the sub-region.

**Figure:**
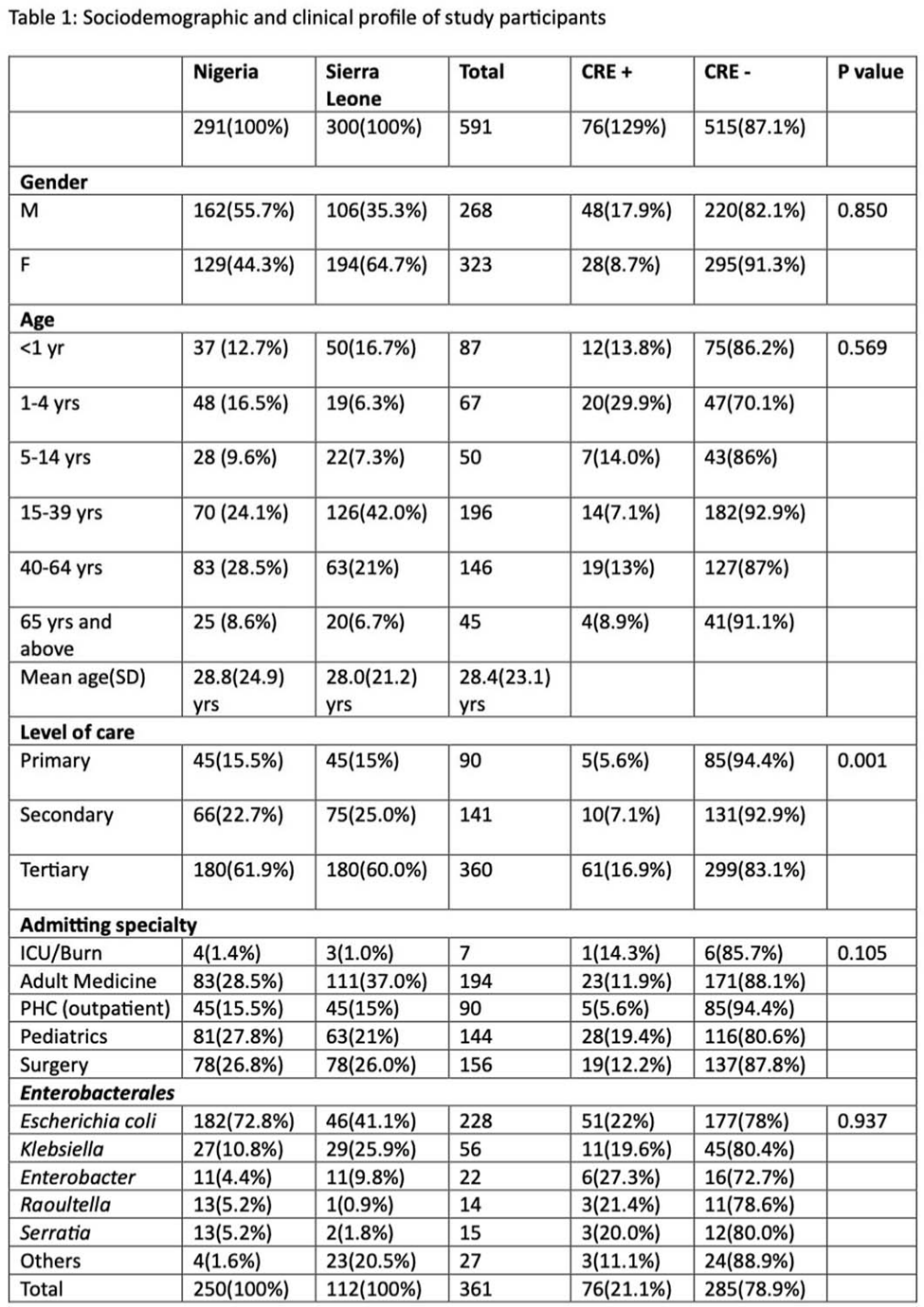

**Figure:**
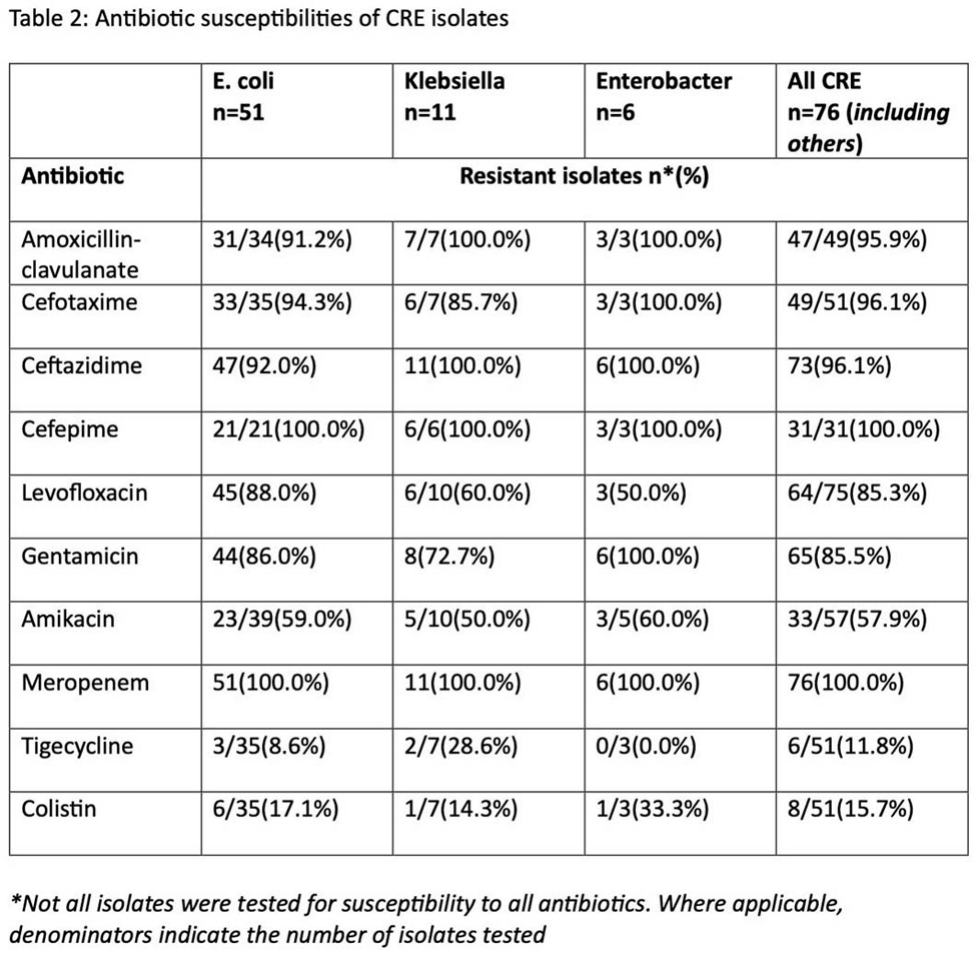

**Methods:**

Patients were screened for CRE by culture of rectal swabs at primary (PHC), secondary (SHC) and tertiary (THC) healthcare facilities in Ibadan, Nigeria and Freetown, Sierra Leone from December 2021 to September 2024. After obtaining consent, demographic and clinical data were collected from patients and their records; and rectal swabs obtained for microscopy culture and sensitivity. Patients with gastrointestinal bleeding and rectal pathology were excluded.

**Results:**

Overall, 591 patients were included, of whom 268(45.3%) were male, 196(33.2%) aged 15-39 years and mean(SD) age was 28.4(23.1) years (Table 1). Ninety (15.2%), 141 (23.9%) and 360 (60.9%) were recruited from PHCs, SHCs and THCs respectively. There were CREs isolated from 76 patients including 51(67.1%) in Nigeria. *E. coli* were most frequently isolated – 51 (65.8%). There were 5(5.6%), 10(7.1%) and 61(16.9%) CRE isolates from PHCs, SHCs and THCs respectively; this difference was significant (*P=0.001*). No CREs isolated from PHCs in Sierra Leone. There were no significant differences in CRE colonization by gender, age, admitting specialty and pathogen. All CRE isolates were resistant to meropenem and cefepime and had at least 95% resistance to the other beta lactams (Table 2). Overall resistance was 11.8% and 15.7% to tigecycline and colistin respectively.

**Conclusion:**

This is the first study to demonstrate CRE rectal colonization at all levels of care in West Africa. There is a need to implement IPC measures within healthcare facilities and communities to prevent their spread. Further studies are needed to understand risk factors for CRE colonization and approaches to effective treatment.

**Disclosures:**

All Authors: No reported disclosures

